# Probiotics and Time to Achieve Full Enteral Feeding in Human Milk-Fed and Formula-Fed Preterm Infants: Systematic Review and Meta-Analysis

**DOI:** 10.3390/nu8080471

**Published:** 2016-07-30

**Authors:** Arianna Aceti, Davide Gori, Giovanni Barone, Maria Luisa Callegari, Maria Pia Fantini, Flavia Indrio, Luca Maggio, Fabio Meneghin, Lorenzo Morelli, Gianvincenzo Zuccotti, Luigi Corvaglia

**Affiliations:** 1 Neonatology and Neonatal Intensive Care Unit, Department of Medical and Surgical Sciences (DIMEC), University of Bologna, S.Orsola-Malpighi Hospital, Bologna 40138, Italy; arianna.aceti2@unibo.it; 2 Task Force on Probiotics of the Italian Society of Neonatology, Milan 20126, Italy; dedegori27@gmail.com (D.G.); gbarone85@yahoo.it (G.B.); marialuisa.callegari@unicatt.it (M.L.C.); mariapia.fantini@unibo.it (M.P.F.); f.indrio@alice.it (F.I.); luca.maggio@fastwebnet.it (L.Ma.); fabio.meneghin@asst-fbf-sacco.it (F.M.); lorenzo.morelli@unicatt.it (L.Mo.); gianvincenzo.zuccotti@unimi.it (G.Z.); 3 Department of Biomedical and Neuromotor Sciences (DIBINEM), University of Bologna, Bologna 40138, Italy; 4 Neonatal Unit, Catholic University, Rome 00168, Italy; 5 Institute of Microbiology, UCSC, Piacenza 29122, Italy; 6 Department of Pediatrics, Aldo Moro University, Bari 70124, Italy; 7 Study Group of Neonatal Gastroenterology and Nutrition of the Italian Society of Neonatology, Milan 20126, Italy; 8 Division of Neonatology, Children Hospital V. Buzzi, ICP, Milan 20154, Italy; 9 Department of Pediatrics, Children Hospital V. Buzzi, University of Milan, Milan 20154, Italy

**Keywords:** probiotics, preterm infants, human milk, full enteral feeding, systematic review

## Abstract

Probiotics have been linked to a reduction in the incidence of necrotizing enterocolitis and late-onset sepsis in preterm infants. Recently, probiotics have also proved to reduce time to achieve full enteral feeding (FEF). However, the relationship between FEF achievement and type of feeding in infants treated with probiotics has not been explored yet. The aim of this systematic review and meta-analysis was to evaluate the effect of probiotics in reducing time to achieve FEF in preterm infants, according to type of feeding (exclusive human milk (HM) vs. formula). Randomized-controlled trials involving preterm infants receiving probiotics, and reporting on time to reach FEF were included in the systematic review. Trials reporting on outcome according to type of feeding (exclusive HM vs. formula) were included in the meta-analysis. Fixed-effect or random-effects models were used as appropriate. Results were expressed as mean difference (MD) with 95% confidence interval (CI). Twenty-five studies were included in the systematic review. In the five studies recruiting exclusively HM-fed preterm infants, those treated with probiotics reached FEF approximately 3 days before controls (MD −3.15 days (95% CI −5.25/−1.05), *p* = 0.003). None of the two studies reporting on exclusively formula-fed infants showed any difference between infants receiving probiotics and controls in terms of FEF achievement. The limited number of included studies did not allow testing for other subgroup differences between HM and formula-fed infants. However, if confirmed in further studies, the 3-days reduction in time to achieve FEF in exclusively HM-fed preterm infants might have significant implications for their clinical management.

## 1. Introduction

Nutrition during critical time windows in early life can affect long-term health [1]. Early provision of optimal enteral nutrition to preterm infants might improve neurodevelopmental outcome by decreasing the rate of several complications of prematurity, such as extrauterine growth restriction, necrotizing enterocolitis (NEC), sepsis, bronchopulmonary dysplasia, and retinopathy of prematurity [2].

Late introduction and slow advancement of enteral feeding may alter gastrointestinal motility and disrupt microbial colonization [3], leading to a delay in establishing full enteral feeding (FEF). The consequent prolonged need for parenteral nutrition can have serious infectious and metabolic complications, which might prolong hospital stay, increase morbidity and mortality, and affect growth and development [4].

Several clinical variables and interventions have been proposed as predictors of the time to FEF achievement in preterm and very-low-birth-weight (VLBW) infants. Among these variables, the influence of type of feeding was also documented, as FEF achievement was delayed in formula-fed infants compared to human milk (HM)-fed infants [5].

Recently, probiotic use has been associated with a reduced time to achieve FEF and better feeding tolerance [6], as well as a reduction of NEC [7,8] and late-onset sepsis [9]. Probiotics are live microorganisms which, when ingested in adequate amounts, confer a health benefit to the host, by modifying the composition and function of gut microbiota and the immunological responses in the host [10]. The role of probiotics in attaining a more rapid achievement of FEF could be related to their favorable effect on the physiological intestinal dysbiosis of preterm infants [11], which is the result of the exposure to a unique environment and to several iatrogenic manipulations, such as broad spectrum antibiotics [12]. It is well known that gut microbiota in HM-fed infants is different compared to formula-fed infants [13]; data from an observational study also suggest a feeding-dependent effect of probiotics, as in that study NEC incidence was reduced in infants treated with probiotics and receiving HM, but not in those exclusively formula-fed [14]. However, the relationship between probiotics and type of feeding in attaining a more rapid achievement of FEF has not been explored yet, even in the most recent meta-analysis on this topic [6].

Thus, the aim of the present paper was to evaluate the effect of probiotics on time to FEF achievement according to type of feeding (exclusive HM vs. formula), by performing a systematic review and meta-analysis of currently available literature on this topic.

## 2. Materials and Methods

### 2.1. Literature Search

The study protocol was designed by the members of the Task Force on Probiotics of the Italian Society of Neonatology. PRISMA guidelines [15] were followed in order to perform a systematic review of published studies reporting the relationship between probiotic use and time to FEF achievement in preterm infants according to type of feeding.

In order to be included in the meta-analysis, studies had to meet the following inclusion criteria: randomized or quasi-randomized clinical trials involving preterm infants (gestational age (GA) <37 weeks) who received, within one month of age, any probiotic compared to placebo or no treatment, and reporting on type of feeding. The outcome of interest was time for FEF achievement (any definition). Only English-written studies and studies involving humans were included in the meta-analysis.

A search was conducted for studies published before 2 March 2016 in PubMed [16], the Cochrane Library [17], and Embase [18]. The following search string was used for the PubMed search: ((preterm infant OR pre-term infant) OR (preterm infants OR pre-term infants) OR (preterm neonate OR pre-term neonate) OR (preterm neonates OR pre-term neonates) OR (preterm newborn OR pre-term newborn) OR (preterm newborns OR pre-term newborns) OR (premature infant OR premature infants) OR (premature neonate OR premature neonates) OR (premature newborn OR premature newborns) OR infant, extremely premature (MeSH Heading (MH)) OR premature birth (MH) OR infant, low birth weight (MH) OR infant, very low birth weight (MH)) AND (full enteral* OR feed*) AND (probiotic OR probiotics OR pro-biotic OR pro-biotics OR probio*)) NOT (animals (MH) NOT humans (MH).

The string was built up by combining all the terms related to probiotics and FEF achievement: PubMed MeSH terms, free-text words, and their combinations obtained through the most proper Boolean operators were used. The same criteria were used for searching the Cochrane Library and Embase.

Arianna Aceti and Luigi Corvaglia performed the literature search: relevant studies were identified from the abstract; full-texts of relevant studies were examined, as well as their reference lists in order to identify additional studies.

### 2.2. Data Extraction and Meta-Analysis

Study details (population, characteristics of probiotic and placebo, type of feeding, and outcome assessment) were evaluated independently by Arianna Aceti and Luigi Corvaglia, and checked by Davide Gori. Study quality was evaluated independently by Arianna Aceti and Davide Gori using the risk of bias tool as proposed by the Cochrane collaboration (Chapter 8 of the Cochrane Handbook of Systematic Reviews) [19].

The corresponding authors of the studies in which days to FEF achievement were not reported as mean ± standard deviation (SD) were contacted by email. When data were not provided, the study was not included in the meta-analysis.

The association between probiotic use and FEF achievement according to type of feeding was evaluated by a meta-analysis conducted by AA and DG using the RevMan software (Cochrane Informatics and Knowledge Management Department, version 5.3.5) downloaded from the Cochrane website [20]. Mean difference (MD) in days to achieve FEF between infants receiving probiotics and those receiving placebo or no treatment was calculated using the inverse variance method, and reported with 95% confidence interval (CI).

For the analysis, we planned to use at first a fixed effect model. Heterogeneity was measured using the *I*^2^ test: if significant heterogeneity was present (*p* < 0.05 from the χ^2^ test) and/or the number of studies was ≤5, a random-effects model was used instead.

## 3. Results

### Literature Search

Overall, 372 papers were identified through the literature search, 155 in PubMed [16], 73 in the Cochrane Library [17], and 144 in Embase [18].

**Figure 1:**
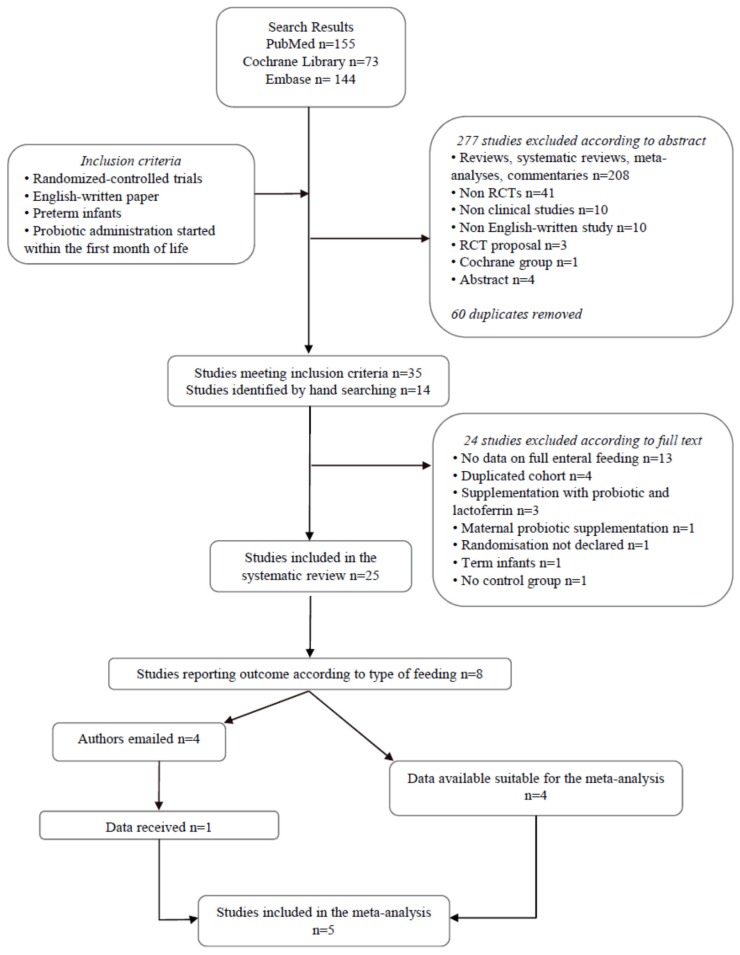
Flow chart of the search strategy used for the systematic review. The relevant number of papers at each point is given.

As shown in Figure 1, 35 studies met the inclusion criteria [21,22,23,24,25,26,27,28,29,30,31,32,33,34,35,36,37,38,39,40,41,42,43,44,45,46,47,48,49,50,51,52,53,54,55]. Fourteen additional papers were identified from the reference lists of included studies or by “snowballing” techniques [52,56,57,58,59,60,61,62,63,64,65,66,67,68].

Twenty-four studies were excluded after examining the full-texts [28,29,31,32,33,35,42,43,44,45,46,47,51,53,54,55,57,58,59,62,63,65,69]. Twenty-five studies were then suitable for inclusion in the systematic review (Table 1) [21,22,23,24,25,26,27,30,34,36,37,38,39,40,41,48,49,50,56,60,61,64,66,68,70].

Among them, only eight studies reported FEF achievement according to type of feeding: infants were fed exclusively HM, either own mother’s (OMM) or donor human milk (DHM), in six studies [22,38,50,56,60,70], while two studies reported FEF in exclusively formula-fed infants [41,61].

The corresponding authors of four of these papers were contacted by email, as data for FEF achievement were not suitable for inclusion in the meta-analysis: mean ± SD of days for FEF achievement were provided for one study [22], while data were unavailable for three studies [41,61,70]; these three studies were thus excluded from the meta-analysis.

Overall, five studies were included in the meta-analysis: in all these studies, infants were fed exclusively HM, either OMM or DHM (Figure 1) [22,38,50,56,60].

**Figure 2:**
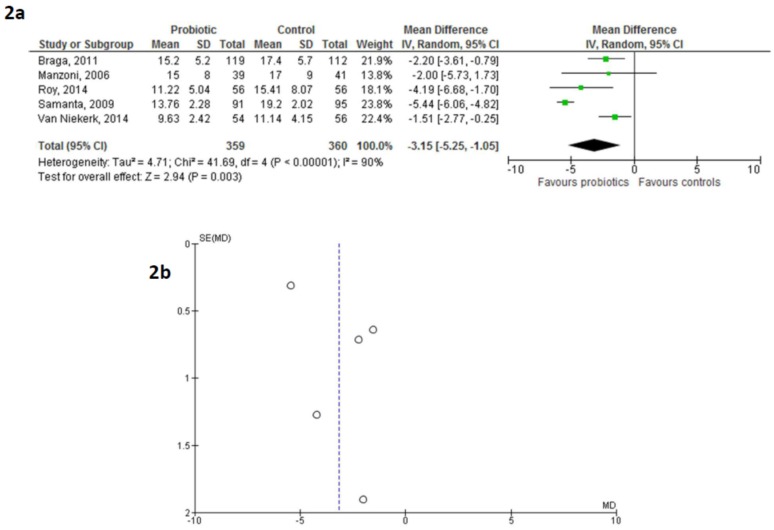
Forest plot (**2a**) and funnel plot (**2b**) showing the association between the use of probiotics and achievement of full enteral feeding in exclusively human milk-fed preterm infants. IV: inverse variance method.

Data from 359 infants in the probiotic group and 360 infants in the control group were evaluated: probiotic use was associated with a reduction in the time for FEF achievement (MD −3.15 days (95% CI −5.25/−1.05), *p* = 0.003; Figure 2a). The funnel plot did not show any clear asymmetry (Figure 2b).

Three studies were not included in the meta-analysis because data on FEF were not available as mean ± SD [41,61,70]. One study reported the use of *Bacillus clausii* in preterm infants with GA < 34 weeks, fed expressed breast milk or DHM [70] and stratified as extreme preterm (GA 27–30 + 6 weeks) and very preterm (GA 31–33 + 6 weeks). In both groups, probiotic use was associated with a reduced time to achieve FEF (risk ratio 0.82 (95% CI 0.74–0.88) and 0.67 (95% CI 0.32–0.77), respectively).

The other two studies reported probiotic use in exclusively formula-fed infants: in the study by Costalos et al., infants born at 28–32 weeks gestation and fed exclusively preterm formula received *Saccharomyces boulardii* or placebo for approximately 30 days [41]. In the study by Stratiki et al., formula-fed infants with a similar gestational age (27–32 weeks) received *Bifidobacterium lactis* vs. no treatment [61]. Neither of these two studies reported any significant difference between groups in terms of time to FEF achievement.

All the studies included in the meta-analysis, except one [50], recruited exclusively infants with birth weight <1500 g. The study by Roy et al. [50] reported specific data for extremely low birth weight (ELBW) infants: time to reach FEF in ELBW infants treated with probiotics was significantly lower than in controls (mean ± SD 13.22 ± 5.04 vs. 17.41 ± 8.07, respectively, *p* = 0.014). None of the studies included in the meta-analysis reported separate data on intrauterine growth restricted (IUGR) infants.

In all the studies, except one [56], a probiotic mix was used: the meta-analysis performed after the exclusion of the study by Manzoni et al., where a single-strain product containing Lactobacillus GG was used, confirmed the results of the overall analysis (MD −3.33 days (95% CI −5.63/−1.04), *p* ≤ 0.004).

## 4. Methodological Study Quality

Evaluation of the quality of the studies included in the meta-analysis according to the risk of bias tool as proposed by the Cochrane Collaboration [19] is shown in Table 2.

## 5. Discussion

The present meta-analysis shows that the use of probiotics in preterm, VLBW infants fed exclusively HM is associated with 3-days reduction in the time to FEF achievement. The only two studies included in the present systematic review in which infants were exclusively formula-fed did not report any difference between the probiotic and the control group.

The single previous meta-analysis investigating FEF as primary outcome showed an overall smaller reduction in the time to FEF achievement, but did not report separate data for HM-fed and formula-fed infants [6]. The studies included in the meta-analysis by Athalye-Jape et al. are almost the same as those included in our systematic review; quite surprisingly, in the majority of the studies included in these two reviews, both HM and formula-fed infants were recruited, but no detailed information on the relationship between type of feeding and outcome was provided.

Type of feeding might modulate the relationship between probiotics and neonatal clinical outcome [14]. It has been previously shown that HM feeding is associated with shorter time to achieve FEF compared to formula feeding [5]. Our meta-analysis, which included only studies where infants were exclusively HM-fed, showed a significant reduction in the time to achieve FEF attributable to probiotics. Despite the limitation given by the small number of studies, a probiotic-related 3-days reduction in time to achieve FEF in preterm infants fed exclusively HM has strong clinical implications and deserves further consideration. When OMM is not available or contraindicated, the use of pasteurized DHM is recommended for preterm infants: pasteurization inactivates most viral and bacterial agents, but at the same time affects some nutritional and immunological properties of HM, including endogenous probiotics [71]. It can be speculated that the beneficial effect of probiotics documented in exclusively HM-fed infants could be attributed to a synergic action exerted by the prebiotic components of HM and the exogenous probiotic, which partially restores the symbiotic properties of naïve HM [72]. In the present meta-analysis, no separate data for OMM-fed and DHM-fed infants were available; for this reason, it is not possible to clarify whether the beneficial effect of HM on FEF achievement applies both to OMM and to DHM.

Heterogeneity among included studies was high; however, given the small number of papers, our ability to explore sources of heterogeneity was limited. In the five included studies, different probiotic strains were used. We aimed to perform strain-specific sub-meta-analyses, in order to clarify whether there was any probiotic product showing a significant benefit in terms of reduction in the time to achieve FEF. However, such analyses were not feasible, as none of the studies used the same probiotic strain or mix. Similarly, it was not possible to explore additional sources of heterogeneity, such as the characteristics of probiotic administration (dose, duration, infant age at probiotic initiation, etc.). In addition, we were unable to test for subgroup differences between HM-fed and formula-fed infants, which might have partially explained the different results in terms of FEF achievement.

Apparently, studies were homogeneous in terms of included populations, as almost all of them recruited only VLBW infants. However, few data on “high-risk” infants, such as ELBW and IUGR infants, could be extrapolated from the main results of the included studies.

The use of probiotics should be weighed against their potential side effects. There are some reports about the occurrence of sepsis in preterm newborns, potentially linked to probiotic administration [73].

However, none of the studies included in the systematic review reported any side effect related to the use of probiotics.

## 6. Conclusions

According to the results of the present meta-analysis, the use of probiotics is linked to 3-days reduction in time to achieve FEF in preterm VLBW infants fed exclusively HM. If confirmed in further studies, this reduction might have strong clinical implications for this high-risk population.

## Figures and Tables

**Table 1:** Studies included in the systematic review.

Author, Year	Study Details	Study Population	Intervention Specie	Milk	Placebo	FEF Definition
			Dose (D)			
			Start of Treatment (S)			
			End of Treatment (E)			
Bin-Nun, 2005 [40]	P	Preterm infants with BW < 1500 g, who began enteral feeding on a weekday	*B. infantis, Str. thermophilus, B. bifidus*	OMM, PFM	HM or FM	100 mL/kg/day
	B		D: 0.35 × 10^9^ CFU each, OD			
	R		S: start of enteral feeding			
	C		E: 36 w postconceptual age			
Braga, 2011 [60]	P	Inborn infants with BW 750–1499 g	*L. casei, B. Breve*	HM (± PFM from w3)	Extra HM	150 mL/kg/day
	DB		D: 3.5 × 10^7^ CFU to 3.5 × 10^9^ CFU OD			
	R		S: day 2			
	C		E: day 30, NEC diagnosis, discharge, death, whichever occurred first			
Costalos, 2003 [41]	P	GA 28–32 w	*Saccharomyces boulardii*	PFM	MDX	Not defined
	R	No major GI problem	D: 1 × 10^9^ CFU BD			
	C	Not receiving antibiotics	S: non-specified			
		Not receiving breast milk	Median duration of probiotic supplementation: 30 days			
Costeloe, 2015 [64]	P	Preterm infants with GA 23–30 + 6 weeks, without any lethal malformation or any malformation of the GI tract	*Bifidobacterium breve BBG-001*	OMM, DHM, FM	Corn starch powder	150 mL/kg/day
	DB		D: 8 · 3–8 · 8 log_10_ CFU/day			
	R		S: as soon as possible after randomisation			
	C		E: 36 w PMA or discharge if sooner			
	Multic.					
Demirel, 2013 [27]	P	Preterm infants with GA ≤ 32 weeks and BW ≤ 1500 g, who survived to feed enterally	*S. boulardii*	HM, FM	None	Not defined
	B		D: 5 × 10^9^ CFU OD			
	R		S: first feed			
	C		E: discharge			
Dilli, 2015 [49]	P	Preterm infants with GA < 32 weeks and BW < 1500 g, born at or transferred to the NICU within the first week of life and fed enterally before inclusion	*B. lactis*	HM, FM	MDX powder	100 mL/kg/day (FEF for hydration)
	DB		D: 5 × 10^9^ CFU			150 mL/kg/day (FEF for growth)
	R		S: beyond d7 after birth			
	C		E: death or discharge (max 8 weeks)			
	Multic.					
Fernández-Carrocera, 2013 [30]	P	Preterm infants with	*L. acidophilus* 1 × 10^9^ CFU/g, *L. rhamnosus* 4.4 × 10^8^ CFU/g, *L. casei* 1 × 10^9^ CFU/g, *L. plantarum* 1.76 × 10^8^ CFU/g, *B. infantis* 2.76 × 10^7^ CFU/g, *Str. thermophilus* 6.6 × 10^5^ CFU/g	OMM, PFM	None	Not defined
	DB	BW < 1500 g	Total D: 1 g powder OD			
	R	Infants with NEC stage IA and stage IB were excluded	S: start of enteral feeding			
	C		E: non specified			
Hays, 2014 [66]	P	Preterm infants with GA 25–31 weeks, BW 700–1600 g, AGA, enteral feeding initiated before day 5	Probiotic group composed of 3 subgroups:	OMM, DM or PFM	MDX	Not defined
	DB	Infants with NEC stage ≥ IB, malformations or severe medical or surgical conditions were excluded	P1 *B. lactis*			
	R		P2 *B. longum*			
	C		P3 *B. lactis + longum*			
	Multic.		D: 1 × 10^9^ CFU each probiotic daily			
			Duration: 4 weeks for infants ≥29 w/6 weeks for infants ≤28 w GA			
Hikaru, 2010 [68]	P	Extremely low birth weight and very low birth weight infants	*B. breve*	OMM, PFM	None	Not defined
	R		D: 0.5 × 10^9^ CFU BD			
	C		S: birth			
			E: discharge from NICU			
Jacobs, 2013 [25]	P	Preterm infants with GA <32 weeks and BW < 1500 g	*B. infantis BB-02* 300 CFU × 10^6^, *Str. thermophilus Th-4* 350 CFU × 10^6^, *B. lactis BB-12* 350 CFU × 10^6^	HM, FM	MDX powder	Enteral feeds of 120 mL/kg for ≥3 days
	DB		Total D: 1 × 10^9^ CFU × 1.5 g MDX powder OD			
	R		S: enteral feed ≥ 1 mL every 4 h			
	C		E: discharge or term corrected age			
	Multic.					
Lin, 2008 [39]	P	Preterm infants with GA < 34 weeks and BW ≤ 1500 g, who survived to feed enterally	*L. acidophilus NCDO 1746, B. bifidum NCDO 1453* 10^9^ CFU	HM, FM	None	Oral intake of 100 mL/kg/day
	B		D: 1 × 10^9^ CFU each probiotic (= 125 mg/kg) BD			
	R		S: day 2 of age			
	C		Duration: 6 weeks			
	Multic.					
Manzoni, 2006 [56]	P	Infants with BW < 1500 g, ≥3 day of life, who started enteral feeding with HM	*L. casei* subspecies *rhamnosus LGG*	OMM, DM	None	Not defined
	DB		D: 6 × 10^9^ CFU/day			
	R		S: day 3 of life			
	C		E: end of the 6th week or discharge			
Mihatsch, 2010 [36]	P	Preterm infants with GA < 30 weeks and BW ≤ 1500 g	*B. lactis BB12*	OMM, PFM	Indistinguishable powder	150 mL/kg/day
	R		D: 2 × 10^9^ CFU/kg 6 times a day			
	C		S: start of enteral feeding			
			E: non specified			
Oncel, 2014 [24]	P	Preterm infants with GA ≤ 32 weeks and BW ≤ 1500 g, who survived to feed enterally	*L. reuteri DSM 17938*	HM, FM	Oil base	Not defined
	DB		D: 1 × 10^8^ CFU OD			
	R		S: first feed			
	C		E: death or discharge			
Patole, 2014 [23]	P	Preterm infants with GA < 33 weeks and BW < 1500 g	*B. breve*	HM, FM	Dextrin	150 mL/kg/day enteral feeding
	DB		D: 3 × 10^9^ CFU OD (1.5 × 10^9^ CFU OD for newborn ≤ 27 w until they reached 50 mL/kg/day enteral feeds)			
	R		S: start of enteral feed			
	C		E: corrected age of 37 w			
Rougé, 2009 [37]	P	Preterm infants with GA < 32 weeks and BW < 1500 g, ≤2 weeks of age, without any disease other than those linked to prematurity, who started enteral feeding before inclusion	*B. longum* BB536, *L. rhamnosus* GG BB536-LGG	OMM, DM or PFM	MDX	Not defined
	DB		Total D: 1 × 108 CFU/day			
	R		S: start of enteral feeding			
	C		E: discharge			
	Bic.					
Roy, 2014 [50]	P	Preterm infants (GA < 37 weeks) and BW < 2500 g, with stable enteral feeding within 72 h of birth	*L. acidophilus* 1.25 × 10^9^ CFU × 1 g, *B. longum* 0.125 × 10^9^ CFU × 1 g, *B. bifidum* 0.125 × 10^9^ CFU × 1 g, *B. lactis* 1 × 10^9^ CFU × 1 g	HM	Sterile water	120 mL/kg/day for ≥3 d
	DB		D: half a 1 g sachet			
	R		S: from 72 h of life			
	C		E: after 6 w or at discharge			
Saengtawesin, 2014 [48]	P	Preterm infants with GA ≤ 34 weeks and BW ≤ 1500 g	*L. acidophilus* 1 × 10^9^ CFU, *B. bifidum* 1 × 10^9^ CFU	HM, PFM	None	150 mL/kg/day
	R		D: 125 mg/kg BD			
	C		S: start of feeding			
			E: 6 w of age or discharge.			
Samanta, 2008 [38]	P	Preterm infants with GA < 32 weeks and BW < 1500 g, who started enteral feeding and survived beyond 48 h of age	*B. infantis, B. bifidum, B. longum,* *L. acidophilus*	HM	None	Not defined
	DB					
	R		D: 2.5 × 10^9^ CFU each probiotic, BD			
	C		S: start of enteral feeding			
			E: discharge			
Sari, 2011 [34]	P	Preterm infants with GA < 32 weeks or BW < 1500 g, who survived to feed enterally	*L. sporogenes*	HM, FM	None	Not defined
	B		D: 0.35 × 10^9^ CFU OD			
	R		S: first feed			
	C		E: discharge			
Serce, 2013 [26]	P	Preterm infants with GA ≤ 32 weeks and BW ≤ 1500 g, who survived to feed enterally	*S. boulardii*	HM, FM	Distilled water	100 mL/kg/day
	M		D: 0.5 × 10^9^ CFU/kg BD			enteral feeding
	R		S: non specified			
	C		E: non specified			
Stratiki, 2007 [61]	P	Preterm infants with GA 27–32 weeks, formula-fed, without major congenital anomalies	*Bifidobacterium lactis*	FM	None	150 mL/kg/day
	B		D: 2 × 10^7^ CFU/g of milk powder			
	R		S: start of enteral feeding			
	C		E: not specified			
Tewari, 2015 [70]	P	Preterm infants with GA < 34 weeks	*Bacillus clausii*	OMM, DHM	Sterile water	180 mL/kg/day
	DB	Excluded if: NEC, congenital anomaly , outborn and >10 days of with sepsis	D: 2.4 × 10^9^ CFU/day			
	R	Stratified as extreme preterm (GA 27–30 + 6) and very preterm (GA 31–33 + 6)	S: by day 5 in asymptomatic and by day 10 in symptomatic infants			
	C		E: 6 weeks of age, discharge or death (whichever occurred first)			
Totsu, 2014 [21]	P	Infants with BW < 1500 g	*B. bifidum*	HM, FM	Dextrin	Postnatal day at which the amount of enteral feeding exceeded 100 mL/kg/day
	DB		D: 2.5 × 10^9^ CFU, divided in two doses			
	CLR		S: within 48 h after birth			
	C		E: body weight 2000 g			
	Multic.					
Van Niekerk, 2014 [22]	P	Preterm infants with GA < 34 weeks and BW < 1250 g, exposed and non-exposed to HIV (only infants unexposed to HIV are included in the meta-analysis)	*L. rhamnosus, B. infantis*	HM	MCT oil	“when infants no longer required the use of IV fluids”
	DB		D: 0.35 × 10^9^ CFU each probiotic			
	R		S: start of enteral feeding			
	C		E: day 28 postconceptual age			

P: prospective; B: blinded; R: randomized; C: controlled; DB: double-blinded; Multic: multicentric; M: masked; CLR: cluster-randomized; BW: birth weight; GA: gestational age; HM: human milk; L.: Lactobacillus; B.: Bifidobacterium; Str.: Streptococcus; S.: Saccharomyces; CFU: colony forming unit; OD: once daily; NEC: necrotizing enterocolitis; BD: twice daily; OMM: own mother’s milk; PFM: preterm formula; FM: formula; MDX: maltodextrin; PMA: postmenstrual age; AGA: appropriate for gestational age.

**Table 2:** Evaluation of the quality of the studies included in the meta-analysis according to the risk of bias tool as proposed by the Cochrane collaboration.

Study	Random Sequence Generation	Allocation Concealment	Blinding	Incomplete Outcome Data	Selective Outcome Reporting	Other Sources of Bias
Braga, 2011 [60]	Low	Low	Low	Low	Unclear	Low
Manzoni, 2006 [56]	Low	Low	Low	Unclear	Unclear	Low
Roy, 2014 [50]	Low	Unclear	Low	Low	Unclear	Unclear
Samanta, 2008 [38]	Low	Low	Low	Unclear	Unclear	Unclear
Van Niekerk, 2014 [22]	Low	Unclear	Low	Unclear	Unclear	Unclear
